# 1988. Early Syphilis in Minnesota: Statewide and Regional Trends, 2018-2022

**DOI:** 10.1093/ofid/ofad500.115

**Published:** 2023-11-27

**Authors:** Elizabeth Dufort, Khalid Bo-Subait, Nicholas Lehnertz, Candy Hadsall, Karmen Dippmann, Cindy Lind Livingston, Ruth Lynfield

**Affiliations:** Minnesota Department of Health, St Paul, MN; Minnesota Department of Health, St Paul, MN; Minnesota Department of Health, St Paul, MN; Minnesota Department of Health, St Paul, MN; Minnesota Department of Health, St Paul, MN; Minnesota Department of Health, St Paul, MN; Minnesota Department of Health, St Paul, MN

## Abstract

**Background:**

Cases of syphilis have been increasing in the US for several years. We assessed the epidemiology of syphilis in MN.

**Methods:**

Syphilis cases are reported to MN Dept of Health through electronic laboratory reporting and are interviewed by disease intervention specialists. We reviewed early syphilis case data (primary, secondary, or early non-primary/secondary) and evaluated demographics, geographic region, HIV infection and substance use.

**Results:**

From 2018-2022 there was a 108% increase in early syphilis with a total of 4,306 cases; 76% males, 65% of whom identified as men who have sex with men (MSM). Median age was 33 (IQR 27-42) years. The average 5-year rates of early syphilis in those who identify as American Indian (AI) (162 per 100,000) and Black, Non-Hispanic (66.9 per 100,00) were 22.2-fold and 9.2-fold higher respectively, compared to White (7.3 per 100,000). 25% of early syphilis cases occurred in persons with HIV (PWH), a 68% increase from 2018-2022 (165 cases in 2018 to 276 in 2022). Substance use was reported in 23% of cases. Cases in females rose 267% (97 in 2018 to 344 in 2022) and in males 81% (478 in 2018 to 866 in 2022). Among early syphilis female cases, 30% were AI and 19% were Black. Congenital syphilis cases also increased from 10 cases in 2018 to 20 in 2022 (rate 15.1 to 31.7 per 100,000 live births).

Across 3 impacted MN regions over the 2018-2022 timeframe, case characteristics varied. In the Twin Cities Region (TCR: n=3154, 73% cases) 81% were male, of whom 73% identified as MSM. In the Duluth Region (DR: n=157, 4% cases), 71% were male of whom 54% identified as MSM. In TCR, 31% cases were PWH and in DR, 13% of cases were PWH. In the Northwest Region (NWR: n=352, 8% cases), 45% were male, of whom 6% identified as MSM. In NWR, 51% cases reported substance use, compared with 20% in TCR and 11% in DR.

**Conclusion:**

Similar to other US areas, syphilis is increasing in MN and is disproportionately impacting AI and Black populations. These trends and the rise in female and congenital cases warrant accelerated public health action. Rather than one statewide syphilis approach, regional differences in epidemiology highlight the need for understanding root challenges, engaging effectively with communities to develop locally specific interventions, access to testing and prenatal screening, and access to care.

**Disclosures:**

**All Authors**: No reported disclosures

